# Racial Identity and Relevance in Teaching Clinical Skills and Diagnostic Medicine: A Small/Pilot Focus Session to Engage Medical Educators

**DOI:** 10.7759/cureus.31288

**Published:** 2022-11-09

**Authors:** Arkene Levy, Jocelyn Mitchell-Williams, Yolanda Payne-Jameau, Ernest Wallace, Chasity O’Malley, Skyler Coetzee, Vijay Rajput

**Affiliations:** 1 Medical Education (Pharmacology), Nova Southeastern University, Dr. Kiran C. Patel College of Allopathic Medicine, Fort Lauderdale, USA; 2 Medical Education, Diversity and Community Affairs, Cooper Medical School of Rowan University, Camden, USA; 3 Medical Education, Nova Southeastern University, Dr. Kiran C. Patel College of Allopathic Medicine, Fort Lauderdale, USA; 4 Medical Education, Cooper Medical School of Rowan University, Camden, USA; 5 Medical Education, Wright State University Boonshoft School of Medicine, Dayton, USA; 6 Medicine, Nova Southeastern University, Dr. Kiran C. Patel College of Allopathic Medicine, Fort Lauderdale, USA

**Keywords:** medical teaching, diagnostic medicine, clinical skills, ethnicity, race

## Abstract

Background

The relevance and importance of using racial and ethnic categories in medical education is an area requiring richer discussion and engagement among all health professions educators.

Objectives

There is a genuine need to identify opportunities for leveraging social and structural determinants of health to address health disparities within medical education. We designed a focus session led by a team of diverse clinical and basic science faculty to analyze how medical curricula can integrate racial/ethnic identity as a clinical indicator. We sought to develop strategies to empower medical students and teachers to integrate race as a social rather than a biological construct.

Methods

The 90-minute focus session included an interactive presentation reviewing the literature related to the use of racial identity in medical education, small group case-based discussions in breakout rooms, a large group debrief, and an optional activity for participants to apply the knowledge gained. The study was approved by the Nova Southeastern University Institutional Review Board (IRB #: 2021-185-NSU).

Results

Thirty-three participants attended the session at the 2021 International Association of Medical Science Educators (IAMSE) Conference. Eleven (33%) participated in both pre-session and post-session surveys. Survey data revealed significant pre-/post-changes in the knowledge of the advantages and disadvantages of using race in medical education. Qualitative data indicated that participants acquired new knowledge related to the integration of racial identity as a clinical indicator and they were willing to incorporate strategies learned into their teaching.

Conclusion

Our study data suggests that this focus session equipped faculty with new knowledge and resources to effectively incorporate racial/ethnic identity into medical school curricula to increase awareness of healthcare disparities.

## Introduction

Race, ethnicity, and their relevance in medical education and the practice of medicine continue to be a topic that is heavily discussed in academia. In the past several years, much emphasis has been placed on the topic at academic conferences, with emphasis on ways to identify and mitigate implicit bias at the forefront. Racial categories and their connection with health equity and healthcare outcomes have been intertwined within the practice of medicine for decades, and this has shaped the way in which knowledge is imparted in medical school curricula, clinical research, and textbooks. There are varying opinions among medical educators, where some are averse to the use of racial categories and race-based guidelines due to concerns that when medical school curricula train students to consider racial categories for patients’ healthcare, this perpetuates systemic issues such as microaggressions and stereotyping [1,2]. On the other hand, some medical educators including physicians of color often support the integration of racial categories into diagnostic medicine and clinical care on the basis that there is value and benefit to patients of color when the aim of racial integration is to highlight social and structural rather than genetic explanations [2-5]. Generally, those in support of leveraging race and ethnicity information for better patient outcomes propose that racial identity can be useful for identifying different disease mechanisms and planning relevant clinical interventions when disease etiology is related to social affiliations and environments [2-5]. This interactive workshop was designed and facilitated by clinical faculty, basic science faculty, and medical students. The overarching goal of the session was to generate a robust discussion around the historical and sociological context of race and ethnicity and openly share ideas and expertise on the best approaches to educating medical students about this topic. This was a focus session presented at the 2021 International Association of Medical Science Educators (IAMSE) Conference. The focus session was led by a team of diverse clinical and basic science faculty with the relevant expertise to direct and stimulate discussions. The specific objectives of the session were to review the literature related to the use of racial identity in medical education, analyze the pros and cons of integrating racial categories into case-based learning and clinical skills and diagnostic medicine teaching, and discuss effective strategies for incorporating racial identity into medical education without misidentifying healthcare needs for vulnerable patients.

## Materials and methods

Ethical approval

The study was approved by the Nova Southeastern University Institutional Review Board (IRB #: 2021-185-NSU).

Participants

Thirty-three (33) conference attendees participated in the focus session on June 16th during the 2021 International Association of Medical Science Educators (IAMSE) Conference. Of the 33 participants, 23 completed the pre-session surveys, 11 completed both the pre-session and post-session surveys, and eight completed the anonymous commitment activity. These participants had diverse backgrounds based on voluntarily self-disclosed demographic information from the pre-post surveys.

Session structure and context

The 90-minute focus session was presented virtually at the 2021 IAMSE Conference, which had the theme of “Global Perspectives on Health Sciences Education.” A didactic literature review-based presentation centered around teaching racial categories in medical education was followed by a small group case-based discussion. Participants worked through cases that promoted discussion on the advantages and disadvantages of integrating racial categories into active learning cases and integrating this information with the social and structural determinants of health. Participants were then brought back to the large group for debriefing and the optional commitment activity. Pre- and post-activity surveys were utilized as a measure of the effectiveness of the presentation and case-based activity by assessing for changes in knowledge and attitudes about racial identity in medical education. Participants were also invited to participate in an anonymous commitment activity. In this activity, participants were prompted to think of an action item that they could implement in the near future to help increase their self-awareness of using racial identity and avoiding microaggressions, based on the examples given in the session and ideas sparked by discussion.

Cases and facilitators

Two short cases with accompanying questions were developed by the research team with the intention of highlighting the use of racial categories in case-based learning. The experiences of the physicians on the research team who were also individuals of color were integrated into the cases to represent realistic scenarios. Four faculty members and a second-year medical student facilitated the small group discussions. Three of the faculty were females who identified as African American, with one being a basic scientist and two being physicians. The fourth physician facilitator and the student were male, and both were persons of color.

Statistical analysis

A paired Student’s t-test was used to assess the statistical significance of the data obtained from the pre- and post-session surveys, with a p-value less than 0.05 being considered statistically significant.

## Results

Demographic characteristics of participants

The demographic characteristics of participants who completed the pre- and post-session surveys are presented in Table 1.

**Table 1:** Demographic characteristics of the participants (n=23) who completed the pre- and post-session surveys

Category	# of respondents (n=23)	% of respondents
Professional title		
Basic science faculty	8	34.78
Physician educator	6	26.09
Other health professions faculty	3	13.04
Administrator	3	13.04
Medical student	2	8.70
Other health professions student	1	4.35
Gender		
Females	19	82.61
Males	3	13.04
Prefer not to answer	1	4.35
Sexual orientation		
Straight or heterosexual	18	78.26
Bisexual	3	13.04
Prefer not to answer	2	8.70
Age (years)		
25-29	2	8.70
35-44	9	39.13
45-54	6	26.09
55-64	4	17.38
65 and over	2	8.70
Race/ethnicity		
Asian	5	21.74
Black, non-Hispanic	1	4.35
Hispanic	3	13.04
White, non-Hispanic	14	60.87

Pre-/post-surveys

**Figure 1:**
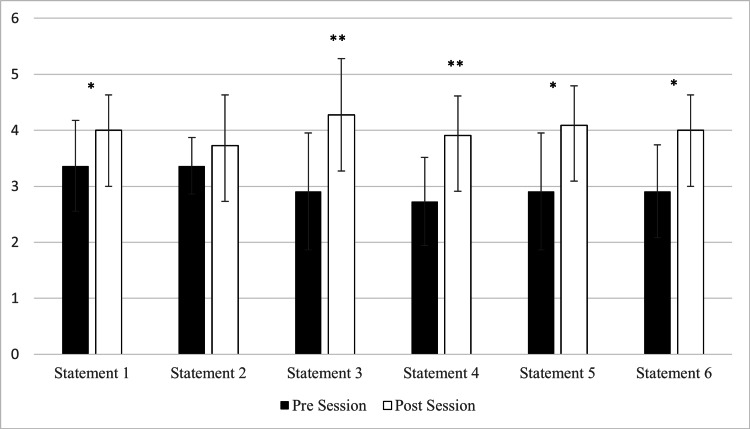
Change in confidence and knowledge Results of the pre- and post-survey responses (n=11) to statements 1-6 as shown in Table 2. Data are represented as the mean value ± the standard deviation. * represents p<0.05; ** represents p<0.01.

For the pre- and post-session surveys (n=11), the participants responded to the six statements shown in Table 2, on a Likert scale of 5 indicating strongly agree, 4 agree, 3 somewhat agree, 2 disagree, and 1 strongly disagree. Participants showed a significantly favorable increase in five of the six statements. Figure 1 depicts the pre- and post-changes for all six statements.

**Table 2:** Pre- and post-survey responses SD: standard deviation

Statement	Mean pre-survey responses (SD)	Mean post-survey responses (SD)	Pre- and post-change P value
Statement 1: “I am confident in my ability to define race and ethnicity”	3.36 (0.81)	4 (0.63)	p=0.026
Statement 2: “I understand and can describe the historical context of race”	3.36 (0.50)	3.73 (0.90)	p=0.341
Statement 3: “I am confident in my ability to discuss race as a biologic versus social construct”	2.91 (1.04)	4.27 (1.01)	p=0.005
Statement 4: “I am confident in my ability to identify advantages of integrating racial categories into medical teaching”	2.73 (0.79)	3.91 (0.70)	p=0.007
Statement 5: “I am confident in my ability to identify disadvantages of integrating racial categories into medical teaching”	2.91 (1.04)	4.09 (0.70)	p=0.011
Statement 6: “I am knowledgeable about effective strategies for incorporating racial identity into medical education”	2.91 (0.83)	4.00 (0.63)	p=0.014

Anonymous commitment activity

Individuals who participated in the voluntary anonymous commitment activity responded with a wide variety of action items. These items reflected responses to the prompt “I would like to commit to the following personal goal regarding the use of racial identity and/or perpetuating racial microaggression in teaching and in my educational interactions.” The collected narrative responses (n=11) are included in Table 3.

**Table 3:** Anonymous commitment activity participant responses eGFR: estimated glomerular filtration rate; CKD: chronic kidney disease; SDOH: social determinants of health

Responses
I will discuss with my curriculum leadership team whether or how to re-incorporate racial self-identity in a consistent and unbiased manner back into patient cases from which that aspect of identity has been removed, to be able to reflect the diversity of our patient population and seed discussions like we have just had.
I will not propagate physician bias and promote stereotypes by the use of outdated, inappropriate question stems or case presentations. I will discuss implicit bias with my students and encourage self-reflection.
I agree with the statement.
I will give my students tools to help them reflect on their implicit bias and use of racial microaggressions in my courses.
I will make sure that the eGFR in our CKD case contains a discussion of the controversy and potential harms of the continued use of race adjustment.
I would like to commit to being open and aware of how our institution’s cases may include content about racial identity that perpetuates racial microaggressions in teaching and alter the cases with the use of the relevant social context of race to promote equity in healthcare.
I will use race and ethnicity for patient advocacy and SDOH.
I will ask questions about the activation of bias or detecting implicit bias in questions, cases, and presentations.

## Discussion

This workshop was intentionally designed to engage medical educators and medical students in the discussion about the use of racial and ethnic categories in medical education. The goal of the activity was to enhance understanding of race-based health disparities and encourage participants to critically analyze the opportunities for appropriate and effective use of racial and ethnic identifiers in medical teaching. There are numerous examples of race-based disparities in medicine that have been refuted in recent years. A traditional example includes the race-based adjustment of spirometer values during pulmonary function testing for Black American patients [6]. Historically, this practice stems from data produced through comparisons of the lungs of Black and White soldiers by physicians during the civil war [1]. However, there is no biological basis for this adjustment as evidenced by recent studies that find no foundation for such corrections [7]. Furthermore, these clinical guidelines place Black Americans specifically at a disadvantage in obtaining disability benefits because of overestimated lung function and might potentially be biasing COVID-19 care by undermining the severity of lung damage and the need for more aggressive recovery treatment plans [8,9]. Other examples of race-based clinical guidelines include the correction of glomerular filtration rate (GFR) for Black Americans and the differential approach to hypertension treatment in Black patients typified by the approval of the drug BiDil (a fixed dose of isosorbide dinitrate plus hydralazine) for the treatment of advanced heart failure in Black patients despite concerns and controversy around the process of its discovery, clinical trials, and marketing [10-13].

Many discussions around race and ethnicity in the medical education literature have focused on the disadvantages of including race and ethnicity in medical decision-making, particularly highlighting that there is significant overlap across racial groups that render race inconsequential for improving diagnosis and care for any one group of individuals [3,14]. Furthermore, the presentation of race in a normalized way in the diagnostic medicine curriculum has been purported to put persons of color at risk for misdiagnoses through racial profiling in the clinical system [2,3].

In this focus session, the participants disclosed through the pre-session survey that there was a need to have more skills and knowledge on how to leverage race and ethnicity information to address the above-highlighted health disparities. Following the didactic session and the case discussions, the participants showed a significant increase in confidence and knowledge about race and medical teaching. This data highlights that sessions of this nature can be beneficial in enhancing the cultural competence of faculty and staff involved in the development and delivery of medical school curricula. This innovative nature of this session also provides a starting point for other medical schools to adapt and customize the presented activities to promote cultural shifts in medical education that will eventually translate into the positive changes that are needed to reduce the systemic barriers faced by individuals of color regarding their healthcare.

Physicians and medical educators are often uncomfortable with discussing race both in preclinical teaching and on the wards, and there is a need for more training for faculty in this regard [15,16]. Population health data categorized by race can be useful for sharpening the diagnostic skills of learners when time is taken to elaborate on the history of racial categories, the concerns around holding race as a biological construct, and the advantages and limits of their utility, especially in the development of case-based learning clinical scenarios that are used routinely in pre-clerkship education [17]. This was highlighted in a recent initiative by the Association of American Medical Colleges (AAMC) to develop “Diversity, Equity, and Inclusion Competencies Across the Learning Continuum,” a framework to help guide faculty development to provide and teach culturally responsive care, including those related to racial disparities [18]. It is therefore imperative that faculty development opportunities are provided for medical educators to enhance their skills and capacity toward addressing race, in order to reduce biases of both learners and faculty [16,19,20].

Limitations

Participation in the session and pre- and post-survey was voluntary with a limited number of participants, which allows for a possible selection bias. However, with the target audience being all faculty in medical education, the representation of the sample should not misrepresent the population as a whole. Although the data was anonymous, it was collected and analyzed by the workshop authors. Demographic information, including professional title, gender, sexual orientation, age, and race/ethnicity, were collected, allowing for analysis of these characteristics for further potentially significant differences. However, as one participant noted, the limited options for providing information about gender and sexual orientation were not inclusive of the spectrum that participants may self-identify on. Opting to use language such as biological sex or separating the two prompts in the future would promote inclusivity.

## Conclusions

The meaningful use of racial categories as a social construct is a timely topic of discussion in medical education and clinical practice. The topic by nature is a sensitive one that might generate passionate responses from both learners and educators. Continued implementation of faculty development sessions of this nature can help medical educators especially to be more informed and confident in their ability to appropriately discuss and utilize racial categories in medical education.
